# Editorial for *Brain Sciences* Special Issue “Advances in Restorative Neurotherapeutic Technologies”

**DOI:** 10.3390/brainsci15030273

**Published:** 2025-03-05

**Authors:** Trenton A. Line, Isabella S. Elkinbard, David A. Purger, Vivek P. Buch

**Affiliations:** 1 Department of Neurological Surgery, Indiana University School of Medicine, Indianapolis, IN 46202, USA; treline@iu.edu; 2 Department of Neurosurgery, Stanford University, Stanford, CA 94305, USA; ielkinba@stanford.edu

## 1. Introduction

From Ramon y Cajal and Golgi’s histological techniques to single-cell RNA sequencing, technological innovations have long driven progress in neuroscience. We are entering an era in which new technologies have the power not only to help us understand the inner workings of the brain, or to treat the symptoms of the diseases that plague it, but to also restore functionality to those affected by disorders of the central nervous system. From individualized network-based neuromodulatory therapies for neurologic and psychiatric disease, to brain–computer interfaces to restore movement in quadriplegics and vision for the blind, cognitive prosthetics for dementia and learning disabilities, seizure-detecting responsive neurostimulators, augmented reality headsets to help neurosurgeons locate brain tumors and wearable electric field-generating devices to help limit their growth, and many more, today’s rich technological landscape is changing the way we think about maintaining and increasing neurological well-being.

This Special Issue explores several more recent technologies that have allowed interdisciplinary teams of clinicians and scientists to revolutionize the approach to treatment-refractory neurologic and psychiatric disease. We examine the latest applications of these techniques, including the potential to not only restore, but also to augment nervous system functioning and improve the quality of life for patients who have exhausted traditional therapies.

Several common themes emerge from the studies covered in this Special Issue, which highlight the technological advancements driving recent trends in neurorehabilitation, neurorestoration, and neuroscience. Multimodal therapies integrating minimally invasive interventions, precision medicine, advanced neuroimaging, functional brain mapping, and neuromodulation combined with interdisciplinarity collaboration allow for optimization and personalization of treatment plans. An evolving understanding of the nervous system’s adaptive responses—and the many ways that these responses can be enhanced or altered through neuromodulation, stem cell therapy, cognitive training, and physical exercise—have allowed us to imagine new horizons for neurological enhancement and recovery.

## 2. Cellular Therapies for Central Nervous System Regeneration

With advancements in recent technologies, the idea of repopulating various cell types in the central nervous system (CNS) that are prone to damage during disease or injury has emerged. The use of stem cells has been a growing area of interest due to the possibility of reprogramming them to differentiate into nearly any cell type. Our increased understanding of stem cell biology has opened the door to applications in previously untreatable conditions. For instance, interest in the use of stem cells in spinal cord injury (SCI) has recently grown due to their ability to increase the expression of neurotrophic factors that supply nutritional value to damaged tissue [1]. The use of stem cells for restoring sensory and functional deficits is also being investigated. In the review, “An Insight into the Prospects and Drawbacks of Stem Cell Therapy for Spinal Cord Injuries: Ongoing Trials and Future Directions”, Khan et al. explored the regenerative potential of a variety of stem cell types, including mesenchymal stromal cells, induced pluripotent stem cells (iPSC), hematopoietic stem cells, human central nervous system stem cells, and embryonic stem cells, as well as various cell delivery methods, including scaffolds, intrathecal injection, and subarachnoid administration. Many of these techniques have demonstrated neurological and functional improvements after SCI [1].

In addition to treating spinal cord injuries, stem cell therapy has been applied towards targeting cortical injuries and Parkinson’s disease. The review by Harary et al. explored the role of stem cell-based therapies in repopulating dopaminergic cells in Parkinson’s disease. Several methods were evaluated, including reintroducing dopaminergic-specific progenitor cells into the striatum, or through tissue-engineered nigrostriatal pathway reconstruction, both of which have demonstrated significant improvements in rodent models and are currently being tested in clinical trials [2]. In addition to Parkinson’s disease, this approach has been utilized in other conditions, such as modulating the immune system in multiple sclerosis [3], slowing down the progression of amyotrophic lateral sclerosis, and reducing huntingtin aggregates and stimulating endogenous neurogenesis in Huntington’s disease [4].

Additionally, in the review, “Cell Replacement Therapy for Brain Repair: Recent Progress and Remaining Challenges for Treating Parkinson’s Disease and Cortical Injury”, Harary et al. delved into the use of iPSC-derived organoids for cortical injury. Organoid technology involves harvesting and reprogramming patient-derived cells to form three-dimensional structures that integrate several cell types, serving as an ideal model system to investigate cortical injury in strokes, as well as a novel therapeutic and regenerative strategy to promote restoration of motor and sensory functions [2,5]. Despite many technical challenges that remain, stem cell technology holds great promise in advancing functional recovery after neurological injury.

## 3. Cellular and Molecular Therapies for Neuronal Circuit Modulation

Since their discovery in the late 19th century, neurons have been the most studied cell type in the central nervous system. In more recent years, glial cells—astrocytes, oligodendrocytes, microglia, and their precursors—have attracted significant attention toward understanding their basic roles in maintaining neuronal functions, enabling rapid propagation of neural signals, and modulating the immune response in the brain [6]. Less well-understood functions of these cell types are being explored as well, including directly modulating circuit activity or interfacing with cancer cells. In the paper “Translating Molecular Approaches to Oligodendrocyte-Mediated Neurological Circuit Modulation”, Song et al. discussed the latest literature around the dynamics of oligodendrocytes, their progenitors, and the myelin sheath they generate and maintain in the CNS, during neurodevelopmental diseases, neuropsychiatric disorders, and brain cancer [6]. The interactions between different glial cell lineages, such as the effect of changes in microglial populations on myelin structure and function, may also contribute to excitatory/inhibitory imbalances seen in several CNS disorders. Novel technologies such as multi-omic data analysis for identifying biomarkers of interest, closed-loop deep brain stimulation, and gene therapy for modulating the function of specific cell types, are all changing the way we understand the complex and intertwined relationship between different cell types within the central nervous system [6].

In addition to cellular approaches, molecular approaches have also been developed for more precise modulation of cells. For example, designer receptors exclusively activated by designer drugs (DREADDS) have been increasingly utilized for understanding and linking specific circuitry with behavioral output in various animal models of psychiatric disease [7]. On the other hand, the incorporation of ultrasound to increase amplification of mechanoluminescent nanotransducers in genetically encoded cellular targets for deep brain stimulation [8] is another notable example of advanced molecular targeting that allows for the precise modulation of certain cell types within the CNS. These innovative technologies are all being used towards molecular reprogramming and cell-type-specific modulation, which ultimately holds great promise in elucidating novel mechanisms by which glial cells function in the CNS. Further research and advancements of these technologies will pave the way towards more precise targeting and modulation of dysfunctional circuitry in a variety of disease states.

## 4. Non-Invasive Electrical and Acoustic Stimulation

Once thought to be largely unchanging beyond the early years of development, the brain possesses a remarkable capacity to adapt its connections in response to various molecular, chemical, electrical, and behavioral stimuli. Several neuromodulation techniques have been shown to enhance neuroplasticity and aid in the process of neurorehabilitation. One such technique is transcranial focused ultrasound (tFUS). In the paper “Clinical Potential of Transcranial Focused Ultrasound for Neurorehabilitation in Pediatric Cancer Survivors”, VanGilder et al. proposed combining tFUS neuromodulation with cognitive training programs for rehabilitation of cognitive functions in pediatric cancer survivors [9].

Survivors of pediatric acute lymphoblastic leukemia (ALL) frequently show deficits in the crucial domains of executive function, processing speed, and attention due to treatment-related effects on the brain during crucial years of development. Cognitive remediation programs targeting attention and working memory in these patients provide lasting improvements in cognitive skills. However, the brain regions affected by cognitive training are variable, limiting the ability to target treatment to an individual’s specific deficits and unique brain organization. Combining cognitive training with tFUS neuromodulation and computer-assisted adaptive learning paradigms could enhance cognitive recovery by augmenting learning-induced neuroplasticity and allowing for precise modulation of relevant brain regions [9].

The authors of this paper discussed tFUS’s applications in brain mapping and neuromodulation and demonstrated its potential to precisely modulate behaviorally relevant, large-scale cognitive networks by targeting subcortical regions that act as relay centers in these networks. They presented promising results from investigations of tFUS neuromodulation for depression, post-stroke cognitive impairment, emotional regulation, and sleep. Finally, they proposed prefrontal or parietal regions as the ideal targets for tFUS in pediatric ALL survivors and suggested that co-registration with neuroimaging could allow for the development of neurofeedback paradigms of cognitive training [9].

In addition, other non-invasive electrical neuromodulation techniques including transcranial electrical stimulation (TES), which includes direct current (tDCS) or alternating current (tACS) stimulation [10], and transcutaneous cranial nerve stimulation were reviewed. These methods offer safe and accessible approaches to neurorehabilitation that can reliably affect cortical activity, induce lasting neuroplastic effects, and produce measurable effects on behavior. TES has shown promise in the treatment of autism spectrum disorder (ASD), attention deficit/hyperactivity disorder (ADHD), Alzheimer’s disease, and mild cognitive impairment [9,11]. Transcutaneous cranial nerve stimulation has shown improved cognition, mood, and sleep in patients with ADHD and improved global cognition, mental health outcomes, and memory in adult cancer survivors [9]. Despite their utility, these methods of electrical stimulation are limited by poor spatial accuracy and their inability to stimulate deep brain regions. tFUS, with its improved accuracy and depth of penetration, may be preferable to noninvasive electrical stimulation going forward.

Several innovative techniques are being developed to further improve tFUS. Researchers are studying the use of genetically encoded sonosensitive ion channels inserted in specific cell types, which can be stimulated via tFUS to noninvasively modulate neurons with high spatiotemporal precision. This method, known as sonogenetics, has been used to control reward circuitry and mitigate symptoms of Parkinson’s disease (PD) in rodents and restore behavioral responses to light in congenitally blind rats [12]. A similar approach—sono-optogenetics—incorporates the enhanced neuronal control provided by optogenetics, but delivers light stimulation noninvasively, sidestepping the major limitation of optogenetics. In this method, tFUS is used to target nanoparticles, which convert sound energy into photons and activate light-sensitive opsins in genetically modified neurons. Recent advancements in nanoparticle design have increased photon yields, allowing for precise, noninvasive stimulation of deep subcortical structures in rodents [8].

Sonogenetic technology combined with wearable, implant-free tFUS devices could potentially be applied to precisely, consistently, and non-invasively deliver neurostimulation to patients throughout their normal day. This could increase the accessibility and utility of neurostimulation treatments for neurologic and psychiatric disorders. A recent study applied a 3D printed wearable FUS device to deliver bilateral stimulation and alleviate motor deficits in a mouse with PD [13]. Wearables are also being developed for recharging implantable TMS systems, which allow for significantly smaller implants that require less invasive implantation [14,15]. Noninvasive and minimally invasive approaches like these hold the potential to radically change how we interface with the brain to enhance healing.

## 5. Assistive Approaches to Restoring Function

While many novel technologies are being applied to directly augment brain functioning and aid in neurorestoration, it is important to consider how factors outside of the brain can be engaged to enhance its adaptive capacities. In their paper “Assisted Cycle Therapy (ACT) Improved Self-Efficacy and Exercise Perception in Middle-Age Adults with Down Syndrome”, Ringenbach et al. presented an interesting discussion of the interplay between exercise, beliefs, perceptions, and neuroplasticity, highlighting the importance of a multimodal approach to optimizing brain health [16].

People with Down syndrome are at increased risk of early-onset Alzheimer disease, which often presents with decreased physical activity in middle-age, making exercise crucial for maintaining their quality of life. This study aimed to increase self-efficacy in middle-aged people with Down syndrome by implementing an assisted cycling regimen. The authors hypothesized that motor assistance while pedaling on a stationary bike would make the exercise experience more positive and, therefore, improve their self-efficacy. Their results suggested that a cycling regimen may improve self-efficacy in patients with down syndrome, but that motor assistance may hinder these improvements. Exercise perception, however, did seem to improve more with assistance than without, indicating that the relationship between exercise perception and self-efficacy may be more complicated than previously thought [16].

The authors reviewed several possible explanations for the relationship between exercise and improved self-efficacy, including a discussion of exercise-associated neuroplasticity, its effects on brain circuits involved in emotional regulation, and how this may have contributed to changes in self-efficacy and exercise perception [16]. Physical exercise has been shown to increase levels of important markers of neuroplasticity including BDNF, IGF-1, and VEGF [17]. Neuroplasticity-based exercise interventions have been applied in the treatment of a variety of neurologic conditions, including Parkinson’s disease, stroke, traumatic brain injury (TBI), and SCI [17,18]. Exercise combined with non-invasive neurostimulation has been proposed to provide synergistic neuroplastic effects, potentially enhancing cognitive outcomes [17].

Cognitive training programs, another assistive approach for augmenting neuroplasticity, have applications beyond the previously discussed use in cancer survivors, demonstrating benefit in rehabilitating cognitive deficits from TBI and neurodegenerative disorders [18,19]. Some of the more innovative approaches to cognitive and physical rehabilitation make use of novel technologies such as brain computer interfaces (BCI), augmented and virtual reality (AR/VR), and robot-assisted exoskeletons to enhance neuroplasticity and expedite recovery [18,19]. BCI technologies can monitor brain activity, communicate with external devices, and provide real-time feedback through stimulation enabling patients to learn to self-regulate their brain activity [20]. BCI neurofeedback therapy has shown promise in motor rehabilitation after stroke and spinal cord injuries, in cognitive rehabilitation, and in the treatment of psychiatric conditions such as anxiety and ADHD [18,20]. Future BCI applications could even be combined with neuroprostheses or robotic exoskeletons to restore motor disabilities [18].

BCI neurofeedback could be further improved by combining it with VR, which offers immersive, personalized, simulated environments that can be adapted based on BCI feedback. VR tools have demonstrated strong neuroplastic effects, can engage specific brain regions, and can enhance a variety of cognitive, motor, and emotional skills [20]. While the full potential of these novel technologies and their limitations have yet to be established, their introduction indicates a promising future for neurorehabilitation.

## 6. Applying Laser Interstitial Thermal Therapy in Multimodal Cancer Treatment

Laser interstitial thermal therapy (LITT) is a minimally invasive thermal ablation technique which has arisen as a potential alternative to resection of brain tumors in patients at high risk for operative morbidity. LITT combined with stereotactic laser probe placement and MRI thermography for real-time feedback is extremely accurate and, in certain cases, comes with fewer risks than traditional resection [21]. However, the utility of LITT in the management of brain tumors extends beyond simple cytoreduction. One such emerging application of LITT was demonstrated by Chung et al. in their paper “Neoadjuvant Chemotherapy with Laser Interstitial Thermal Therapy in Central Nervous System Neuroblastoma: Illustrative Case and Literature Review” [22]. The authors described the case of a 4-year-old girl who presented with a large, highly vascular tumor located in the left frontotemporal lobe, later diagnosed as CNS neuroblastoma. Given the patient’s young age, and the tumor’s size, vascularity, and location near critical deep structures, resection carried a high risk of neurological morbidity. Further, potentially aggressive tumors such as CNS neuroblastoma can be challenging to treat with traditional resection and chemotherapy.

Chung et al. utilized new evidence linking thermal ablation with a multifaceted tissue reaction that can be combined with other therapies to synergize their effects. LITT causes disruption of the blood–brain barrier for up to 4–6 weeks, a property that is being leveraged to enhance drug delivery to the CNS and potentially improve the efficacy of systemic chemotherapies [23,24]. Additionally, the inflammatory response, hyperthermia, and increased blood–brain barrier permeability induced by LITT are thought to augment local immune function, enhance the endogenous antitumor response, and improve the efficacy of immune checkpoint inhibitors [24,25]. In the young 4-year old, applying precise molecular profiling and leveraging the synergistic effects of LITT, chemotherapy, and radiation, a favorable outcome was achieved and the risks of resection were avoided. At one year follow-up, the patient was doing well with no evidence of recurrence [22].

From combining noninvasive neuromodulation with computer-assisted cognitive remediation programs, to using LITT to enhance drug delivery and antitumor response, advancements in minimally invasive technologies have transformed the landscape of neurotherapeutics. While minimally invasive techniques may have limitations on their own compared to more invasive approaches, they can be implemented in a multimodal regimen to synergize their beneficial effects and minimize risk exposure.

## 7. Conclusions

This Special Issue provides a glimpse into the wide array of innovative neurotherapeutic technologies that continue to expand our understanding of the human nervous system’s capacity to adapt and heal. Stem cells can be engineered to repopulate specific damaged or diseased cell types, or to create organoids for restoring cortical structures damaged by stroke or injury. A growing body of research surrounding oligodendrocytes, oligodendrocyte precursor cells, and microglia has prompted the creation of new therapies targeting these cell types, including gene therapies, closed loop deep brain stimulation, DREADDS, optogenetics, and sonogenetics. Non-invasive neuromodulation, advanced neuroimaging, cognitive training, exercise, computers, and machines are being combined in creative regimens to enhance neuroplasticity and aid in cognitive rehabilitation. At the cutting edge of neurotherapeutics, interdisciplinary teams of specialized clinicians and scientists collaborate to combine advanced diagnostic and therapeutic technologies, synergizing their effects to offer highly personalized, low-risk cancer treatment. Today’s rich technological landscape and our growing understanding of the nervous system have paved the way for solutions to problems long thought to be unsolvable, changing the way we think about neurological recovery.

## Abbreviations

The following abbreviations are used in this manuscript:

DOAJ	Directory of open access journals
CNS	Three letter acronym
SCI	Spinal Cord Injury
iPSC	Induced Pluripotent Stem Cells
DREADDS	Designer Receptors Exclusively Activated by Designer Drugs
tFUS	Transcranial Focused Ultrasound
ALL	Acute Lymphoblastic Leukemia
TES	Transcranial Electrical Stimulation
tDCS	Transcranial Direct Current Stimulation
tACS	Transcranial Alternating Current Stimulation
ASD	Autism Spectrum Disorder
ADHD	Attention Deficit Hyperactivity Disorder
PD	Parkinson Disease
ACT	Assisted Cycle Therapy
TBI	Traumatic Brain Injury
BCI	Brain Computer Interface
AR/VR	Augmented Reality/Virtual Reality
LITT	Laser Interstitial Thermal Therapy

## References

[1] Khan S.I., Ahmed N., Ahsan K., Abbasi M., Maugeri R., Chowdhury D., Bonosi L., Brunasso L., Costanzo R., Iacopino D.G. (2023). An Insight into the Prospects and Drawbacks of Stem Cell Therapy for Spinal Cord Injuries: Ongoing Trials and Future Directions. Brain Sci..

[2] Harary P.M., Jgamadze D., Kim J., Wolf J.A., Song H., Ming G.-L., Cullen D.K., Chen H.I. (2023). Cell Replacement Therapy for Brain Repair: Recent Progress and Remaining Challenges for Treating Parkinson’s Disease and Cortical Injury. Brain Sci..

[3] Nawar A.A., Farid A.M., Wally R., Tharwat E.K., Sameh A., Elkaramany Y., Asla M.M., Kamel W.A. (2024). Efficacy and safety of stem cell transplantation for multiple sclerosis: A systematic review and meta-analysis of randomized controlled trials. Sci. Rep..

[4] Cecerska-Heryć E., Pękała M., Serwin N., Gliźniewicz M., Grygorcewicz B., Michalczyk A., Heryć R., Budkowska M., Dołęgowska B. (2023). The Use of Stem Cells as a Potential Treatment Method for Selected Neurodegenerative Diseases: Review. Cell. Mol. Neurobiol..

[5] Jgamadze D., Lim J.T., Zhang Z., Harary P.M., Germi J., Mensah-Brown K., Adam C.D., Mirzakhalili E., Singh S., Gu J.B. (2023). Structural and functional integration of human forebrain organoids with the injured adult rat visual system. Cell Stem Cell.

[6] Song J., Saglam A., Zuchero J.B., Buch V.P. (2024). Translating Molecular Approaches to Oligodendrocyte-Mediated Neurological Circuit Modulation. Brain Sci..

[7] Whissell P.D., Tohyama S., Martin L.J. (2016). The Use of DREADDs to Deconstruct Behavior. Front. Genet..

[8] Wang W., Kevin Tang K.W., Pyatnitskiy I., Liu X., Shi X., Huo D., Jeong J., Wynn T., Sangani A., Baker A. (2023). Ultrasound-Induced Cascade Amplification in a Mechanoluminescent Nanotransducer for Enhanced Sono-Optogenetic Deep Brain Stimulation. ACS Nano.

[9] VanGilder P., Tanner J., Krull K.R., Sitaram R. (2024). Clinical Potential of Transcranial Focused Ultrasound for Neurorehabilitation in Pediatric Cancer Survivors. Brain Sci..

[10] Zhang S., Qin Y., Wang J., Yu Y., Wu L., Zhang T. (2023). Noninvasive Electrical Stimulation Neuromodulation and Digital Brain Technology: A Review. Biomedicines.

[11] Chen R., Huang L., Wang R., Fei J., Wang H., Wang J. (2024). Advances in Non-Invasive Neuromodulation Techniques for Improving Cognitive Function: A Review. Brain Sci..

[12] Wu P., Liu Z., Tao W., Lai Y., Yang G., Yuan L. (2024). The principles and promising future of sonogenetics for precision medicine. Theranostics.

[13] Hu Z., Yang Y., Yang L., Gong Y., Chukwu C., Ye D., Yue Y., Yuan J., Kravitz A.V., Chen H. (2024). Airy-beam holographic sonogenetics for advancing neuromodulation precision and flexibility. Proc. Natl. Acad. Sci. USA.

[14] Alrashdan F.T., Chen J.C., Singer A., Avants B.W., Yang K., Robinson J.T. (2021). Wearable wireless power systems for ‘ME-BIT’ magnetoelectric-powered bio implants. J. Neural Eng..

[15] Woods J.E., Singer A.L., Alrashdan F., Tan W., Tan C., Sheth S.A., Sheth S.A., Robinson J.T. (2023). Millimeter-sized battery-free epidural cortical stimulators. medRxiv.

[16] Ringenbach S.D.R., Arnold N.E., Tucker K., Rand M.K., Studenka B.E., Ringenbach S.B., Chen C.-C. (2023). Assisted Cycle Therapy (ACT) Improved Self-Efficacy and Exercise Perception in Middle-Age Adults with Down Syndrome. Brain Sci..

[17] Evancho A., Tyler W.J., McGregor K. (2023). A review of combined neuromodulation and physical therapy interventions for enhanced neurorehabilitation. Front. Hum. Neurosci..

[18] Kumar J., Patel T., Sugandh F., Dev J., Kumar U., Adeeb M., Kachhadia M.P., Puri P., Prachi F., Zaman M.U. (2023). Innovative Approaches and Therapies to Enhance Neuroplasticity and Promote Recovery in Patients with Neurological Disorders: A Narrative Review. Cureus.

[19] Zotey V., Andhale A., Shegekar T., Juganavar A. (2023). Adaptive Neuroplasticity in Brain Injury Recovery: Strategies and Insights. Cureus.

[20] Drigas A., Sideraki A. (2024). Brain Neuroplasticity Leveraging Virtual Reality and Brain–Computer Interface Technologies. Sensors.

[21] Chen C., Lee I., Tatsui C., Elder T., Sloan A.E. (2021). Laser interstitial thermotherapy (LITT) for the treatment of tumors of the brain and spine: A brief review. J. Neuro-Oncol..

[22] Chung J.E., Iqbal O., Krishnan C., Harrod V., Tyler-Kabara E., Lu R.O., Ho W.S. (2023). Neoadjuvant Chemotherapy with Laser Interstitial Thermal Therapy in Central Nervous System Neuroblastoma: Illustrative Case and Literature Review. Brain Sci..

[23] Bakrbaldawi A.A.A., Zhu Z., Zheng Z., Zhu J., Jiang H. (2024). An Overview of MR-Guided Laser Interstitial Thermal Therapy (MRg-LITT) in Disrupting the Blood-Brain Barrier: Efficacy and Duration. J. Integr. Neurosci..

[24] Lerner E.C., Edwards R.M., Wilkinson D.S., Fecci P.E. (2022). Laser ablation: Heating up the anti-tumor response in the intracranial compartment. Adv. Drug Deliv. Rev..

[25] Chandar J.S., Bhatia S., Ingle S., Mendez Valdez M.J., Maric D., Seetharam D., Desgraves J.F., Govindarajan V., Daggubati L., Merenzon M. (2023). Laser Interstitial Thermal Therapy Induces Robust Local Immune Response for Newly Diagnosed Glioblastoma with Long-term Survival and Disease Control. J. Immunother..

